# Numerical investigation on 2D viscoelastic fluid due to exponentially stretching surface with magnetic effects: an application of non-Fourier flux theory

**DOI:** 10.1007/s00521-016-2832-4

**Published:** 2017-02-01

**Authors:** S. Bilal, M. Y. Malik, M. Awais, Khalil-ur-Rehman, Arif. Hussain, I. Khan

**Affiliations:** Department of Mathematics, Quaid-i-Azam University, Islamabad, 44000 Pakistan

**Keywords:** Cattaneo–Christove heat flux model, Casson fluid model, Exponentially stretching sheet, Shooting method

## Abstract

Two-dimensional flow of Casson fluid toward an exponentially stretched surface in view of Cattaneo–Christove flux theory is discoursed in current communication. Flow pattern within boundary layer under the effectiveness of magnetic field is also contemplated in the communication. Non-dimensionalized governing expressions are attained through transformation procedure. To anticipate the fascinating features of present work, solution of resulted nonlinear differential system is computed with the collaborated help of shooting scheme and Runge–Kutta method. The influence of involved variables on velocity and temperature fields is scrutinized. Contribution of thermal relaxation is explicitly pointed out. Evaluation of convective heat transfer and friction factor in the fluid flow is visualized through graphs and tables. Additionally, the assurance of present work is affirmed by developing comparison with previous findings in the literature which sets a trade mark for the implementation of numerical approach. It is inferred from the thorough examination of the analysis that present formulation reduces to classical Fourier’s problem by considering $\varLambda = 0$. Furthermore, decreasing pattern in temperature distribution is depicted in the presence of Cattaneo–Christove flux law as compared to heat transfer due to the Fourier’s law.

## Introduction

Recent advancement in modern technology has fascinated the attention of researchers toward the study of heat transfer phenomenons. Therefore, the investigation of heat transfer characteristics in various imperative situations has gained admirable physical significance due to their valuable utility in energy production, nuclear reactor cooling, biomedical applications, etc. Fourier did inaugural work by presenting parabolic equations for the description of temperature of flow field and has draw back that these parabolic equations describes the small disturbance throughout the medium. Therefore, many researchers tried their best to amend the classical Fourier’s law. Among these researchers, Cattaneo [1] successfully modified the law by encompassing material invariant derivative. In their tremendous theoretic exploration, they introduced thermal relaxation time which makes the nature of temperature equation to be hyperbolic and more realistic in describing the temperature of fluid. Tibullo and Zampoli [2] described the significance of such type of heat transportation phenomenon. They addressed that such transportation has remarkable physical prominence in many processes expanding its span from nanotechnology to the modeling of skin burn injury. Christov [3] replaced time derivative in Maxwell–Cattaneo law by Oldryod upper convected derivative. Convective heat transport by considering energy equation of Cattaneo type was evaluated by Straughan [4]. Straughan [5] presented the impact of velocity slip on coupled flow for newly proposed Cattaneo heat flux equation. They reported that thermal relaxation time reduces the temperature of fluid flow.

The flow transit problems arisen due to stretching sheets have superficial role in daily life phenomenons and utilized pervasively in various engineering processes. Specifically, these are useful in synthetic architect such as paper production, aerodynamic extrusion of sheets, drawing of plastics, cooling of metallic sheets in baths and many others. Currently, researchers have anticipated through several experimental studies that desired output from industrial objects can be attained by varying velocity in different ways. After deep surveys, they came up with the decision that velocity may be sinusoidal, exponential or nonlinear. In the process of annealing and thinning of copper wires, we achieve excellent quality of product by considering exponential velocity distributions. Thus, the analyst and engineers at present have paid remarkable attention toward the mechanics of boundary layer flow with exponential velocity distribution. For this purpose, several thought provoking studies have conducted by researchers, but for sake of brevity we have mentioned few. Magyari and Keller [6] did phenomenal work by scrutinizing thermo-physical aspects of viscous fluid over an exponentially stretched surface. They adopted numerical and analytical procedure to interpret the exponential variation in temperature distribution. Bhattacharrya and Vejravelu [7] addressed the steady boundary layer flow of viscous fluid with reactive mass transfer over an exponentially varying stretched surface. They conducted computational analysis of problem by employing Rk-5 method. He concluded that mass transfer rate increments for mounting values of stretching ratio parameter. Elbashbeshy et al. [8] probed numerical solution for description of heat and flow pattern in an ambient medium driven by an exponential surface. Mukhopaday [9] considered thermally stratified flow of Newtonian fluid toward an exponential surface. He explored that heat transfer rate increases in thermally stratified medium. Bidin [10] incorporated radiative heat flux in Newtonian fluid induced by exponentially variable sheet. They extracted results from their investigation that radiation causes inclination in thermal transfer of fluid and Prandtl number tends to decline the temperature of flow. El-Aziz [11] studied the contribution of buoyancy forces in attendance of dissipative effects on micropolar flow over an exponentially stretching configuration. Ishak et al. [12] reconnoitered the influence of magnetic and radiation effects on viscous flow over an exponential stretching sheet. He recommended that coefficient of heat transfer tends to decrease for increasing values of radiation and magnetic parameter.

It is noted that investigators *have shown* remarkable interest toward the analysis of non-Newtonian mechanics because of their eminence in daily life processes, chemical processes and industrial processes. Due to such exemplary importance, non-Newtonian fluids are divided in to shear thinning, shear thickening and viscoelastic fluids. Among these, non-Newtonian fluids describing the characteristics of viscoelastic fluids grade two, grade three and power law fluid models are proposed. However, Casson fluid model is one of the fittest model which describes the properties of viscoelastic fluid model in more realistic way than other models. Due to overwhelming valuability of viscoelastic materials, researchers have proposed numerous studies regarding Casson fluid model. Paramanik [13] inspected the behavior of non-Newtonian fluids by considering Casson fluid along with effects of radiation. During his investigation, he assimilated the effects of suction and blowing on fluid flow. He perceived during the study that Casson fluid enhances the temperature of fluid flow. He also noticed that thermal radiation increases the thermal diffusivity of fluid which enhances the temperature of fluid flow. The inaugural work on this rheological model was done by Casson [14]. He developed flow equation for pigment oil-suspensions of printing ink. Nadeem et al. [15] investigated Casson fluid flow over exponentially shrinking sheet. They found that by increasing Casson fluid parameter velocity distribution of fluid flow increases and similar behavior is observed for increasing value of shrinking parameter. Animasuan et al. [16] parametrically studied the influence of variable thermo-physical properties on Casson fluid over exponentially stretching sheet. They revealed through their investigation that variable plastic dynamic viscosity parameter of Casson fluid corresponds to an increase in the velocity profiles and decline behavior is analyzed in temperature throughout the boundary layer. Nadeem et al. [17] probed solution of Casson fluid flow over three-dimensional stretching surface. They showed through their study that Casson fluid parameter suppresses velocity in both lateral directions. Malik et al. [18] investigated hyperbolic tangent fluid flow over stretching cylinder in the presence of magnetic field. They implemented Keller box scheme to attain desired results. Nadeem et al. [19] inspected the effects of magnetic field on Casson fluid flow over an exponentially shrinking surface. They computed numerical solution of the problem by using shooting method. They found that magnitude of velocity and boundary layer thickness reduces for increasing values of Casson fluid parameter. Blood flow analysis of Prandtl fluid through tapered stenosed arteries surrounded by permeable walls was discussed by Ellahi et al. [20]. They computed approximate solution by implementing perturbation technique and sketched stream lines to interpret the flow behavior. Effects of Hall and ion slip on magnetohydrodynamic flow of Jeffery fluid enclosed by non-uniform rectangular-shaped duct were probed by Ellahi et al. [21]. They performed analysis on peristaltic transport under the constraints of low magnetic Reynold’s number and long wavelength assumptions. The steady boundary layer flow of Burger fluid near stagnation point toward linear stretching surface was anticipated by Hayat et al. [22]. The influential role of Newtonian heating on stagnant flow of Burger fluid was done by Hayat et al. [23]. In order to interpret the pattern of fluid flow, analytical solution was presented via Homotopy analysis method. Hayat et al. [24] conducted comparative study to explore the effects of heat generation/absorption and Newtonian heating on Powell Eyring fluid. Alsaedi et al. [25] carried out analysis to investigate mass transfer in Burger fluid flow in the attendance of first-order chemical reaction. They instituted that retardation time in Burger’s fluid enhances the magnitude of flow. Thermo-physical characteristics of Burger fluid in the presence of Joule heating and magnetic field was manifested by Awais et al. [26].

Electrically conducting flows, which respond to imposition of magnetic fields, have received relatively significant considerations. The study of magnetohydrodynamic (MHD) flow is of valuable interest in modern metallurgical and metal working processes. Some important applications include MHD accelerators, power generators systems and cooling of nuclear reactors. Malik et al. [27] studied MHD flow of tangent hyperbolic fluid over stretching cylinder. They addressed that the velocity profile and skin friction is decreasing function of Hartmann number. Akbar et al. [28] investigated Eyring Powell fluid flow over stretching sheet with magnetic field effects. They reported that Eyring Powell parameter decreases for increasing values of intensity of magnetic parameter. Mabood et al. [29] inspected nanofluid flow over stretching sheet with magnetic effects. They explored through their inspection that velocity suppresses and temperature enhances for increasing values of magnetic parameter. Salahuddin et al. [30] manipulated their investigation to study the impact of magnetic field and variable thermal conductivity on tangent hyperbolic fluid flow with exponentially varying viscosity. They found that for increasing values of magnetic parameter, skin friction increases and velocity of fluid flow decreases. Malik et al. [31] examined numerical solution of MHD stagnation point flow of Williamson fluid. During their exploration, they instituted that Weissenberg number *We* and magnetic parameter *M* cause declination in fluid velocity. On the one hand, the mentioned parameters tend to increase the thermal transport. Malik et al. [32] explicated their findings on mixed convection flow of MHD Eyring Powell fluid over a stretching sheet. They explored that velocity profile enhances by increasing curvature parameter and fluid parameter whereas it decays for increasing value of magnetic parameter. Haq et al. [33] anticipated the influence of magnetic field on stagnant boundary layer flow of viscous nanofluid in the attendance of thermal radiation and slip effects. Numerical solution of bionic peristaltic flow of pseudoplastic fluid enclosed by asymmetric channel was addressed by Khan et al. [34]. Zeeshan et al. [35] illuminated the effects of magnetic dipole on the flow of ferromagnetic fluid toward stretching surface. They formulated momentum and energy expressions involving the role of ferromagnetic particles. Afterward, they attained numerical solution of the constructed problem to investigate the effects of involved parameters. Rashidi et al. [36] worked on the impact of transversely applied magnetic field on two-dimensional fluid flow. They considered diamond-shaped obstacle to investigate heat transfer characteristics in the fluid domain. They established that effects of transverse magnetic field are more significant as to the application of streamwise magnetic field. Enhancement in heat and mass transfer features of peristaltic flow with the inclusion of carbon nanoparticles under the effects of magnetic field was elucidated by Akbar et al. [37]. They measured increasing trend in current density for mounting values of magnetic Reynold’s number. Collaborated effects of ferrohydrodynamic and magnetohydrodynamic fields on nanofluid surrounded by vessel were elaborated by Akbar et al. [38]. They adopted Boltzmann procedure to highlight the effect of pertinent parameters on flow field. Their attained results show that magnetic field considerably decreases the velocity, whereas enhances the skin friction factor. Kandelousi and Ellahi [39] evaluated peristaltic flow with the implication four different types of nanoparticles. They concluded that mass flux in the fluid flow escalates by utilizing various natured nanoparticles. Nadeem et al. [40] studied mass transport in three-dimensional water-based nanofluid driven by exponentially stretched surface and adopted numerical procedure to depict the variation in flow fields within related boundary layers. Nadeem et al. [41] addressed boundary layer flow of viscous fluid due to unsteady shrinking surface. Adomian method was utilized to obtain the solution of requisite differential equations.

The purpose of present study is to explore the results for Casson fluid flow over exponentially stretching sheet with Cattaneo–Christov heat flux model. During the investigation, focus is made on modified energy equation proposed by Cattaneo which is modified form of Fourier’s law of heat conduction. Effects of pertinent parameters involved in energy equation including fluid relaxation time are also presented graphically and numerically. To the best of our knowledge, this is totally new examination because no one has considered Cattaneo flux model with Casson fluid. In this article, behavior of viscoelastic fluid model with thermal relaxation time effects are deliberated through graphs and tables.

## Mathematical formulation

**Fig. 1:**
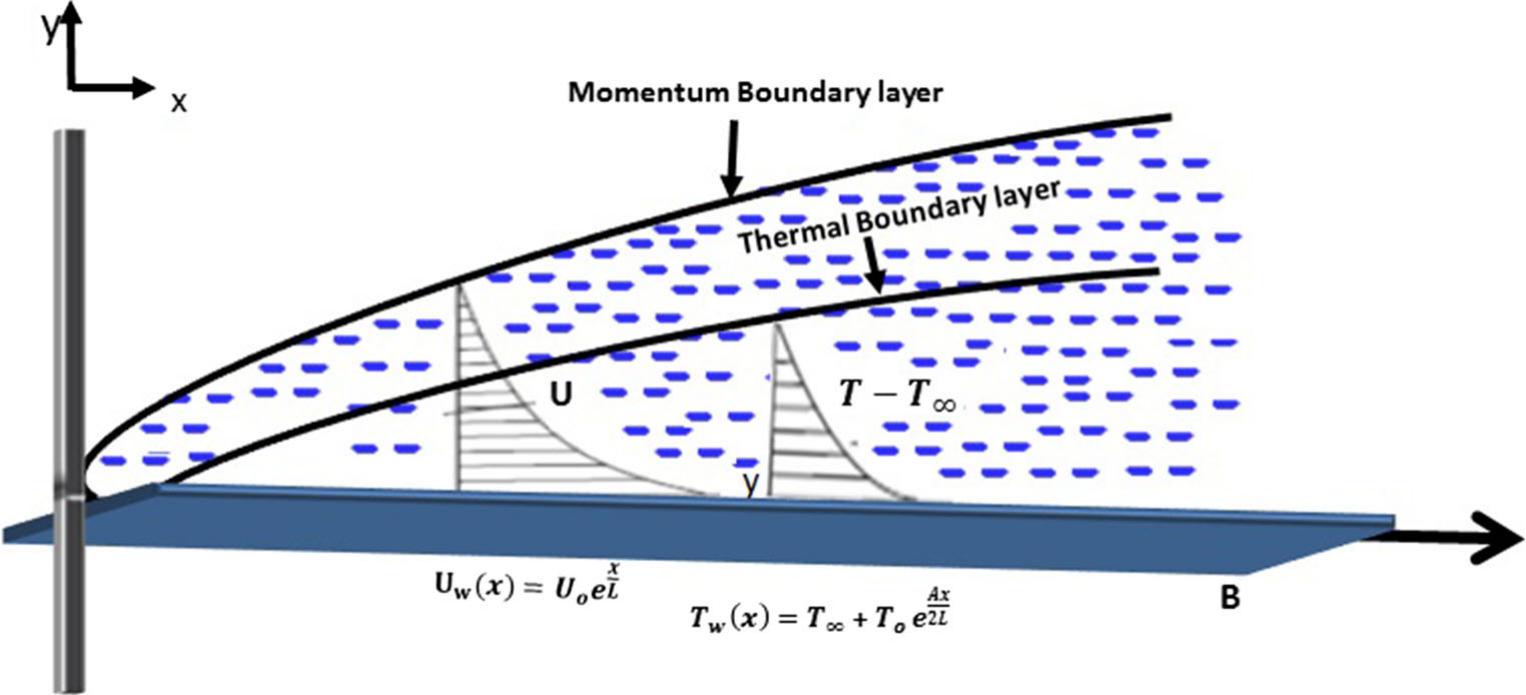
Physical interpretation of geometry

Consider two-dimensional steady incompressible boundary layer flow of Casson fluid over a exponentially stretching sheet. The fluid flow is confined to $y>0.$ Two equal forces are applied along *x*-axis, so that wall is stretched keeping origin fixed as shown in Fig. 1.

The rheological equation for Casson fluid problem is as follows

1 $$\tau _{ij}= {} 2\left( \mu _{\beta }+\frac{p_{\mathrm{y}}}{\sqrt{2\pi }}\right) e_{ij}\quad \pi >\pi _{\mathrm{c}},$$

2 $$\tau _{ij}= {} 2\left( \mu _{\beta }+\frac{p_{\mathrm{y}}}{\sqrt{2\pi _{\mathrm{c}}}}\right) e_{ij}\quad \pi <\pi _{c},$$

where $\pi =e_{ij}e_{ij}$ and $e_{ij}$ are the (*i*, *j*)th component of deformation rate, $\pi$ is product of component of deformation with itself, $\pi _{\mathrm{c}}$ is critical value of this product based on the non-Newtonian model, $\mu _{\beta }$ is dynamic viscosity of the non-Newtonian fluid model and $p_{\mathrm{y}}$ is yield stress of fluid flow. The sheet is stretched in its own plane with velocity $U_{\mathrm{w}}(x)= U_{\mathrm{o}}e^{\frac{x}{L}}$ and variable surface temperature of the form $T_{\mathrm{w}}=T_{\infty }+T_{\mathrm{o}}e^{\frac{Ax}{2L}},$ where $T_{\mathrm{o}}$ denotes the heating/cooling reference temperature. Here $U_{\mathrm{o}}$ is constant and *L* is reference length. It is important to mention that exponential velocity at wall is $U_{\mathrm{o}}e^{\frac{x}{L}}$ is valid for $x \ll L$. It is found that when $x\ge L$ the effect of exponential velocity on wall is shy rocket.

After utilizing boundary layer approximations continuity, momentum and energy equations are described as follows

3 $$\frac{\partial u}{\partial x}+\frac{\partial v}{\partial y}=0,$$

4 $$u\frac{\partial u}{\partial x}+v\frac{\partial u}{\partial y}=\nu \left( 1+\frac{1 }{\beta }\right) \frac{\partial ^{2}u}{\partial y^{2}}-\frac{\sigma B^{2}}{\rho }u,$$

5 $$\rho c_{\mathrm{p}}\left( u\frac{\partial T}{\partial x}+v\frac{\partial T}{\partial y} \right) =-\nabla \cdot q,$$

where *u* and *v* are velocity components along *x*- and *y*-directions, $\nu$ is kinematic viscosity, $\beta =\frac{\mu _{\beta }\sqrt{2\pi _{\mathrm{c}}}}{p_{\mathrm{y}}}$ is Casson fluid parameter, *T* is temperature of local fluid and *q* is heat flux satisfies relation [5].

6 $$q+\lambda \left( \frac{\partial q}{\partial t}+V \cdot \nabla q-q \cdot \nabla V+(\nabla \cdot V)\cdot q\right) =-k\nabla T,$$

in which $\lambda$ is relaxation time for heat flux, *V* is velocity vector, *k* is thermal conductivity and defined as the property of a material to conduct heat. It is evaluated primarily in terms of Fourier’s Law for heat conduction and mathematically expressed as $\alpha \rho c_{\mathrm{p}}=k,$ where $\alpha$ is thermal diffusivity, $\rho$ is density of fluid and $c_{\mathrm{p}}$ is specific heat at constant pressure. Eliminating *q* from Eqs. (4) and (5), we get the relation,

7 $$\begin{aligned}&u\frac{\partial T}{\partial x}+v\frac{\partial T}{\partial y}+\lambda \left[ \left( u\frac{\partial u}{\partial x}+v\frac{\partial u}{\partial y}\right) \frac{ \partial T}{\partial x}+\left( u\frac{\partial v}{\partial x}+v\frac{\partial v}{ \partial y}\right) \right. \nonumber \\&\left. \quad \frac{\partial T}{\partial y}+u^{2}\frac{\partial ^{2}T}{ \partial x^{2}}+v^{2}\frac{\partial ^{2}T}{\partial y^{2}}+2uv\frac{\partial ^{2}T}{\partial x\partial y}\right] =\alpha \frac{\partial ^{2}T}{\partial y^{2}}. \end{aligned}$$

By introducing the following transformations.

8 $$\begin{aligned} &\eta= {} \sqrt{\frac{U_{0}}{2l\nu }}e^{\frac{x}{2L}}y, \nonumber \\ &u= {} U_{\mathrm{o}}e^{\frac{x}{L}}f^{\prime }\quad ,v=-\sqrt{\frac{\nu U_{0}}{2l}} e^{\frac{x}{2L}}\left( f+\eta f^{\prime }\right) , \nonumber \\ &\theta (\eta )= {} \frac{T-T_{\infty }}{T_{\mathrm{w}}-T_{\infty }}. \end{aligned}$$

Equation (3) is satisfied, whereas the Eqs. (4)–(7) reduces to:

9 $$\left( 1+\frac{1}{\beta }\right) f^{\prime \prime \prime }+ff^{\prime \prime }-2f^{\prime 2}-Mf^{\prime }=0,$$

10 $$\begin{aligned}&\frac{1}{Pr}\theta ^{\prime \prime }+f\theta ^{\prime }-Af^{\prime }\theta +\frac{\varLambda }{2}\left[ Aff^{\prime \prime }\theta -A(2+A)f^{\prime 2}\theta \right. \nonumber \\&\left. \quad +(1+2A)ff^{\prime }\theta ^{\prime }-f^{2}\theta ^{\prime \prime }\right] =0, \end{aligned}$$

under transformed boundary conditions

11 $$\begin{aligned} &f(0)= {} 0,\quad f^{\prime }(0)=1,\quad \theta (0) =1, \nonumber \\ &f^{\prime }(\infty )\rightarrow {} 0,\quad \theta (\infty ) \rightarrow 0. \end{aligned}$$

where $\varLambda =\frac{\lambda U_{\mathrm{o}}e^{\frac{x}{L}}}{L}$ is non-dimensional thermal relaxation time parameter, $Pr =\frac{\nu }{k}$ is the Prandtl number, $M=\frac{2\sigma B^{2}L}{\rho U_{\mathrm{o}}}$ is magnetic parameter and *A* is temperature exponent parameter. It is important to mention that $\varLambda =0$ corresponds to classical Fourier law of heat conduction. If we substitute $\varLambda =0$ in Eq. (10), we get the temperature equation describing Fourier law of heat conduction.

The physical quantities skin friction coefficient and local Nusselt number are defined as

12 $$C_{\mathrm{f}}=\frac{\tau _{w}}{\rho \left( U_{\mathrm{o}}\exp \left( \frac{x}{2L}\right) \right) ^{2}},\quad Nu_{x}= \frac{xq_{\mathrm{w}}}{k\left( T_{\mathrm{w}}-T_{\infty }\right) },$$

where $\tau _{\mathrm{w}}$ is known as shear stress or skin friction along stretching sheet

13 $$\begin{aligned} &\tau _{\mathrm{w}}= {} \mu _{\mathrm{b}}\left( 1+\frac{1}{\beta }\right) \left( \frac{\partial u}{\partial y} \right) _{y=0}, \nonumber \\ &q_{\mathrm{w}}= {} -k\left( \frac{\partial T}{\partial y}\right) _{y=0}. \end{aligned}$$

$q_{\mathrm{w}}$ is known as a heat flux from sheet.

The skin friction coefficient and local Nusselt number in dimensionless form are

14 $$\begin{aligned} &\sqrt{2}C_{\mathrm{f}}Re_{x}^{1/2}= {} \left( 1+\frac{1}{\beta }\right) f^{\prime \prime }(0), \nonumber \\ &Nu_{x}Re_{x}^{-1/2}= {} -\theta ^{\prime }(0). \end{aligned}$$

where $$Re_{x}^{1/2}=\frac{U_{\mathrm{o}}L}{\nu }.$$

## Numerical scheme

In the present analysis, boundary layer flow of Casson fluid accompanied by heat transfer toward an exponentially stretched surface has been developed. The constructed nonlinear partial differential equations are metamorphosed in to ordinary differential equations by employing similarity transformations. Since the locally similar coupled ordinary differential equations are highly nonlinear so for better analysis of solution shooting technique with Runge–Kutta method is applied. The variations in model for different values of influential parameter are also discussed. To solve any differential equation by Runge–Kutta–Fehlberg method, there are some elementary steps. Initial step is to transform the nonlinear ordinary differential Eqs. (9) and (10) along with boundary condition in Eq. (11) in to the system of first-order equations by utilizing substitutions $(y_{1},y_{2}, y_{3}, y_{4},y_{5})=(f,\,f^{\prime },\,f^{\prime \prime },\theta ,\theta ^{\prime })$. This substitution yields.

15 $$\begin{aligned}&\left( \begin{array}{c} y_{1}^{\prime } \\ y_{2}^{\prime } \\ y_{3}^{\prime } \\ y_{4}^{\prime } \\ y_{5}^{\prime } \end{array} \right) =\left( \begin{array}{c} y_{2} \\ y_{3} \\ y_{3}^{\prime }=\frac{2y_{2}^{2}-y(1)y_{3}+My_{2}}{\left( 1+\frac{1}{\beta }\right) } \\ y_{5} \\ \frac{\left( -y_{1}y_{5}+Ay_{2}y_{4}-\frac{\varLambda }{2} \left( Ay_{1}y_{3}y_{4}-A(2+A)y_{2}^{2}y_{4}+(1+2A)y_{1}y_{2}y_{5}\right) \right) }{\left( \frac{1}{ Pr}-\frac{\varLambda }{2}x_{1}^{2}\right) } \end{array} \right) . \end{aligned}$$

16 $$\begin{aligned}&\left( \begin{array}{c} y_{1}(0) \\ y_{2}(0) \\ y_{2}(\infty ) \\ y_{4}(0) \\ y_{4}(\infty ) \end{array} =\left( \begin{array}{c} 0 \\ 1 \\ 0 \\ 1 \\ 0 \end{array} \right) \right) \end{aligned}$$

**Fig. 2:**
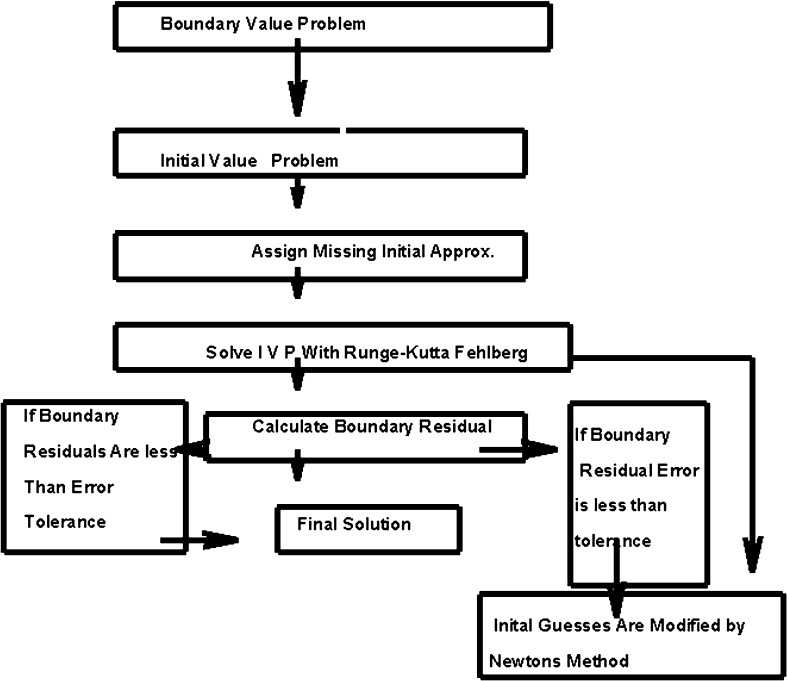
Schematic diagram of shooting method

Now, for better physical interpretation of problem, we require five initial conditions corresponding to Eq. (15). But Eq. (16) has only three initial conditions. Thus, for further proceeding, we have to find missing conditions. For this purpose, we select $y_{3}(0) = 1, y_{5}(0) = -1$ and found that the approximation is excellent for physical insight of problem. Now, solution process is started for computation of fluid velocity and temperature. The process of solution is terminated if absolute difference between given and computed boundary conditions, i.e., $y_{2}(\infty )$ and $y_{4}(\infty )$ is less than tolerance error. But on the other hand, if this difference is larger than tolerance error the initial guesses are refined through Newton method (Fig. 2).

## Results and discussion

**Fig. 3:**
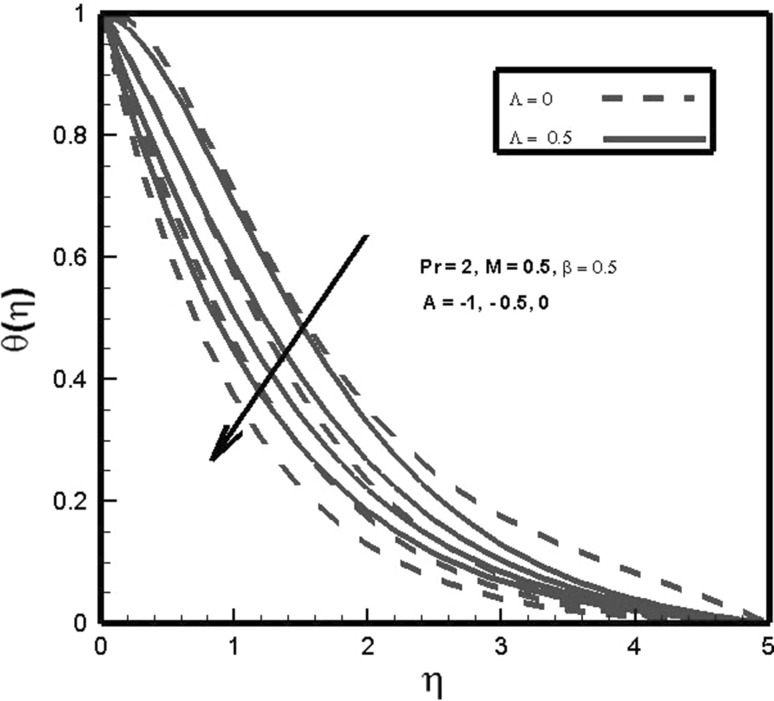
Effects of *A* and $\varLambda$ on $\theta (\eta )$

This section is presented to captivate the focus of researchers by exploring the impact of adopted parameters on transport equations. Firstly, the requisite partial differential equations under the frame work of Bousiqueness approximation are transmuted into nonlinear ordinary differential equation by implementing appropriate transformations. These developed equations are solved computationally by applying shooting method. The influence of emerging dimensionless variables on momentum, thermal fields, the skin friction factor and convective heat transfer is exhibited in graphical manner. The accuracy of present results is tested with existing literature and an excellent agreement is found (Fig. 3).

Table 1 is presented to investigate the fluctuation in the behavior of Nusselt number for increasing values of thermal relaxation parameter $\varLambda$, exponential stretching parameter *A* and Prandtl number *Pr* on Nusselt number. It is exhibited that Nusselt number increases for increasing values of temperature exponent parameter *A*. The reason behind the increment in the values of Nusselt number is due to the fact that temperature exponent parameter *A* increases the motion of fluid molecules which increases thermal energy of fluid particles; as a consequence, heat energy is transferred from the surface. It is also manifested in the table that for increasing values of thermal relaxation parameter $\varLambda ,$ coefficient of convective heat transfer increases. It can be justified physically by the fact that increments in the values of temperature exponent parameter *A* increase the slope of wall temperature, thus the heat transfer from the surface increases. Effect of Prandtl number *Pr* on heat transfer coefficient is also explored. It is noticed that thermal boundary layer thickness reduces with an increase in Prandtl number *Pr*.

**Table 1:** Numerical variation of $\varLambda$, *A* and *Pr* on $-\theta ^{\prime }(0)$

$\varLambda$	*A*	*Pr*	$-\theta ^{\prime }(0)$
		2	0.94411
0.5	0.5	3	1.28730
		4	1.61625
	0.2		0.94411
0.5	0.4	2	1.11950
	0.6		1.2939
0.2			1.07150
0.4	0.5	0.2	1.16140
0.6			1.2540

Table 2 describes the influence of magnetic parameter *M* and Casson fluid parameter $\beta$ on skin friction coefficient. It is instituted that increase in both parameters tends to increase in skin friction coefficient for fluid flow. Magnetic parameter *M* causes inclination in skin friction coefficient due to the fact that it enhances the resistive force which lessens the velocity of fluid flow; as a result, skin friction forces become dominant. It is also observed that Casson fluid parameter $\beta$ suppresses the velocity of fluid. This variation is true physically because by increasing Casson parameter $\beta$ yield stress falls and consequently momentum boundary layer thickness increases.

**Table 2:** Numerical variation of *M* and $\beta$ on $C_{\mathrm{f}}Re_{x}^{\frac{1}{2}}$

*M*	$\beta$	$-\left( 1+\frac{1}{\beta }\right) f^{\prime \prime }(0)$
1		0.9451
2	0.5	1.1044
3		1.2464
	0.5	0.9451
1	1	1.1523
	2	1.3303

Table 3 is presented for the accuracy of numerical results for Nusselt number with previous literature survey presented by Magyari and Keller [6], Bidin [10], El-Aziz [11], Ishak et al. [12] and Paramanik [13]. Similar behavior is found for increasing values of Prandtl number *Pr* on heat transfer coefficient as displayed in previous literature survey. It is also originated that along with similarity in behavior, results are also in total agreement with each other.

**Table 3:** Comparison of Nusselt number with variation in *Pr*

*Pr*	Magyari and Keller [6]	Bidin [10]	El-Aziz [11]	Ishak et al. [12]	Pramanik [13]	Present results
1	0.9458	0.9547	0.9458	0.9458	0.9547	0.9531
2	–	1.4147	–	1.4715	1.4714	1.4624
3	1.8961	1.8961	1.8961	1.8961	1.8961	1.8959
4	2.5001	–	2.5001	2.5001	2.5001	2.5001

Figure 3 displays the impact of temperature exponent parameter *A* and thermal relaxation parameter $\varLambda$ on temperature profile. It is found that temperature distribution is a decreasing function of both mentioned parameters. For negative values of temperature exponent, reverse heat flow is expected in vicinity of surface, whereas with an increase in values of temperature exponent *A* wall slope of temperature function increases sharply. Similarly, temperature inversely relates to thermal relaxation parameter $\varLambda$, so it declines the temperature of fluid flow. For $\varLambda =0$ the Cattaneo–Christov law is reduced to Fourier law of heat conduction.

**Fig. 4:**
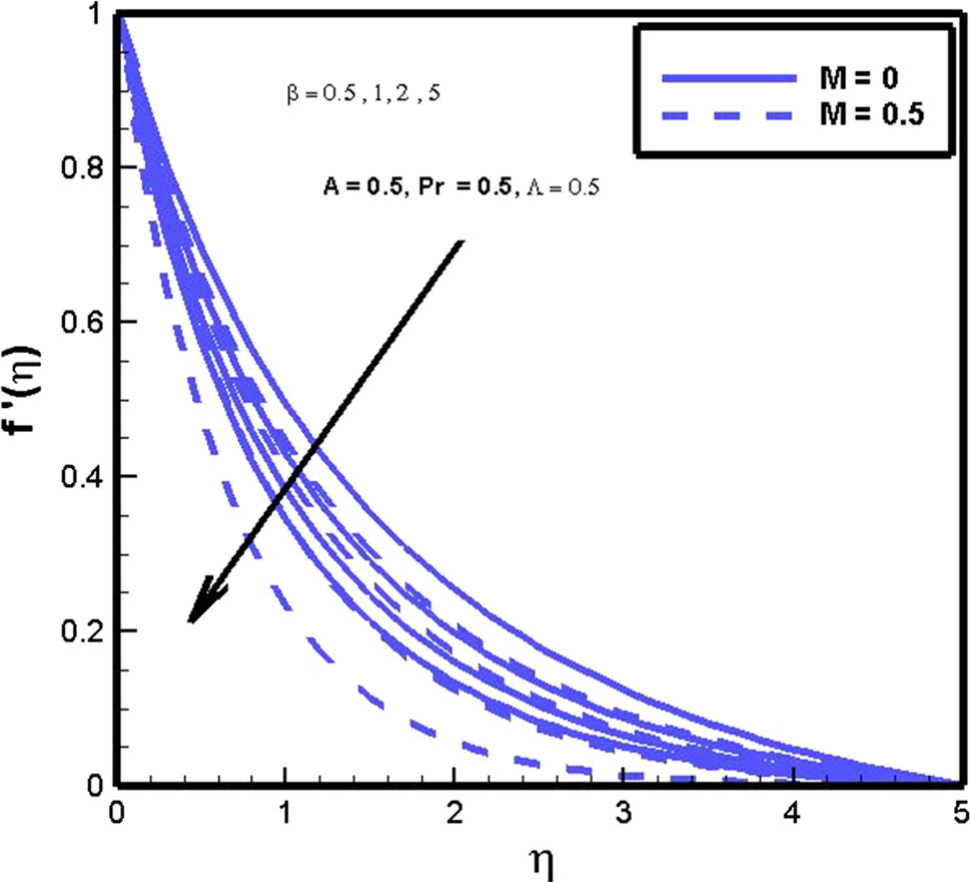
Effects of $\beta$ and *M* on $f^{\prime }(\eta )$

Effects of Casson fluid parameter $\beta$ on velocity profile for $M=0$ (hydrodynamic case) and $M\ne 0$ (magnetohydrodynamic) are exhibited in Fig. 4. It is depicted through the displayed figure that for increasing values of magnetic parameter *M* and Casson fluid parameter $\beta$ velocity of fluid distribution decreases. This effect physically happens due to the fact that transverse magnetic field induces drag in terms of Lorentz force which opposes motion of fluid and rate of transport is considerably reduced. Casson fluid parameter $\beta$ suppresses the velocity of fluid as demonstrated. The cause for declination in velocity is due to the reason that by enhancing Casson fluid parameter $\beta$ yield stresses falls due to fall down of yield stress fluid particle after deformation cannot retain their original position; as a result, velocity of fluid particle reduces. It is also worth mentioning fact that present problem reduces to Newtonian fluid flow for $\beta =\infty.$

**Fig. 5:**
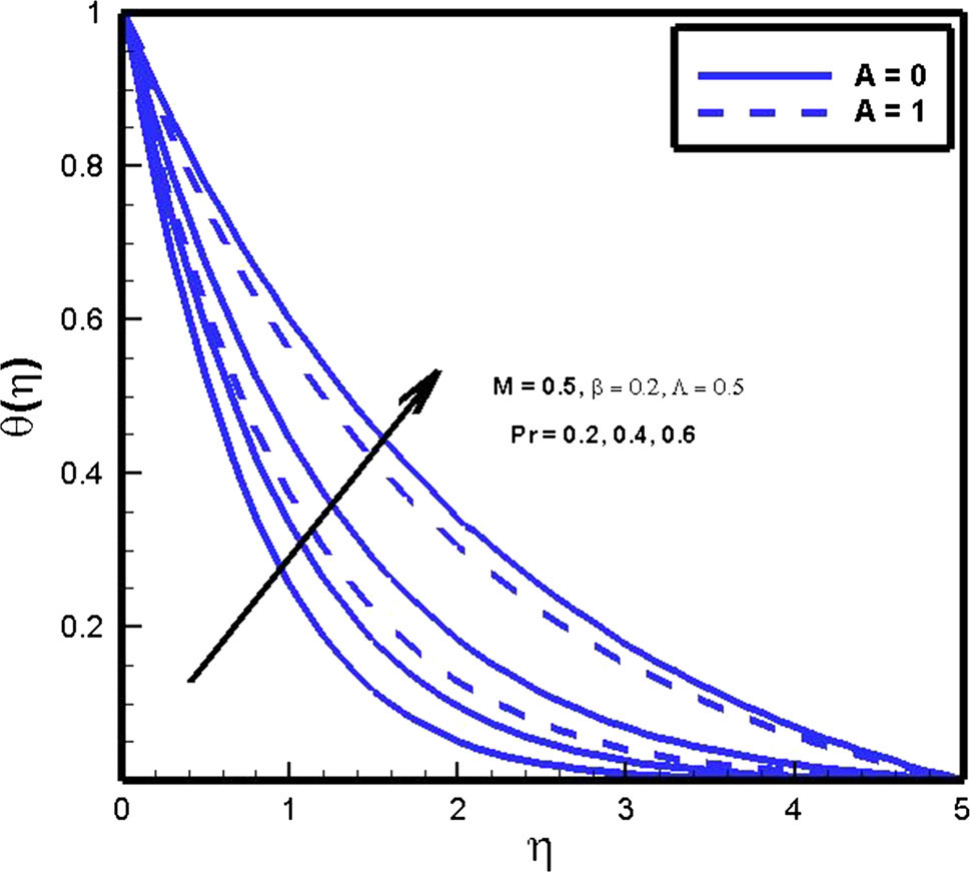
Effects of *Pr* and $\varLambda$ on $\theta (\eta )$

Figure 5 illustrates the sway of Prandtl number *Pr* and temperature exponent parameter *A* on temperature profile and it is found that by increasing Prandtl number *Pr*, temperature of fluid flow decreases. The reason for the behavior is that Prandtl number *Pr* signifies the ratio of viscous diffusion to thermal diffusion, so by increasing Prandtl number *Pr* viscous diffusion increases as result temperature of fluid decreases.

**Fig. 6:**
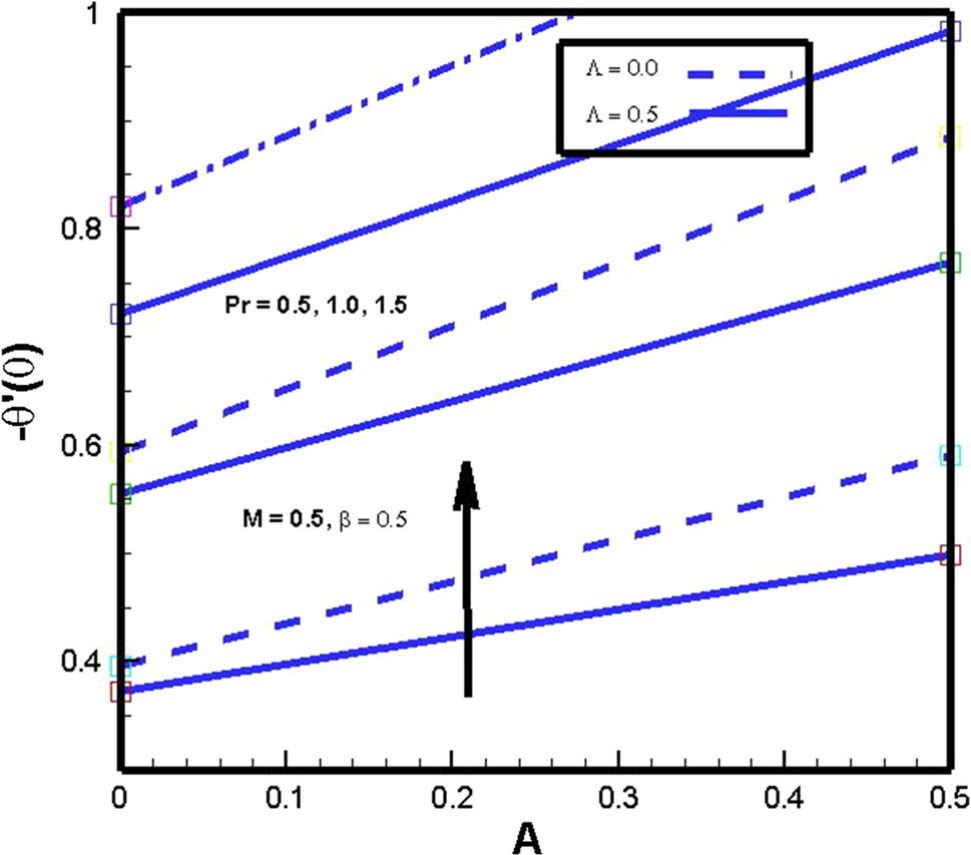
Effects of *Pr* and $\varLambda$ on $-\theta ^{\prime }(0)$

Figure 6 shows the behavior of Prandtl number *Pr*, temperature exponent parameter *A* and thermal relaxation parameter $\varLambda$ on Nusselt number. Nusselt number is found to be increasing function of temperature exponent parameter *A*. As values of temperature exponent increase, more kinetic energy is transferred to fluid molecules which enhances the heat transfer rate. It is also explored that by enhancing values of thermal relaxation parameter $\varLambda$ heat transfer rate increases. This inclination is caused due to the statistics that as thermal relaxation $\varLambda$ inversely relates to temperature of fluid which results in decrease in thermal boundary layer thickness. In response to this behavior, heat transfer rate increases in order to maintain the temperature of fluid flow. The impact of Prandtl number on heat transfer coefficient is also depicted. It is found that coefficient of convective heat transfer increases for increasing values of Prandtl number *Pr*. It holds practically since *Pr* is the ratio of momentum to thermal diffusivity. Thus, by increasing *Pr*, momentum transport accelerates which enhances convective heat transfer and declines conductive heat transfer.

## Conclusions

A theoretical investigation of Casson fluid flow with Cattaneo–Christov heat flux model is executed. The resulting partial differential equations were transformed to a set of coupled ordinary differential equations and solved numerically by utilizing shooting technique. Graphical and tabular mode of computed results is presented to illustrate the details of the heat transfer characteristics and their dependence on physical parameters appearing in the formulated problem.

The present work leads to the following specific conclusion.

- Temperature profile decreases for increasing values of thermal relaxation parameter $\varLambda$ and temperature exponent parameter *A*.
- For higher values of Casson fluid parameter $\beta$ and magnetic parameter *M*, the velocity profile reduces.
- Wall temperature gradient decreases for increasing values of thermal relaxation parameter $\varLambda$ whereas opposite behavior is depicted for temperature exponent parameter *A* and Prandtl number *Pr*.
- Skin friction is higher for larger values of Casson parameter $\beta$ and magnetic parameter *M*.
- Coefficient of convective heat transfer depreciates in the presence of Cattaneo–Christove heat law due to the inclusion of thermal relaxation parameter.
